# Marine versus Non-Marine Bacterial Exopolysaccharides and Their Skincare Applications

**DOI:** 10.3390/md21110582

**Published:** 2023-11-07

**Authors:** Fanny Benhadda, Agata Zykwinska, Sylvia Colliec-Jouault, Corinne Sinquin, Bertrand Thollas, Anthony Courtois, Nicola Fuzzati, Alix Toribio, Christine Delbarre-Ladrat

**Affiliations:** 1 Ifremer, MASAE Microbiologie Aliment Santé Environnement, F-44000 Nantes, France; fanny.benhadda@chanel.com (F.B.); sylvia.colliec.jouault@ifremer.fr (S.C.-J.); corinne.sinquin@ifremer.fr (C.S.); christine.delbarre.ladrat@ifremer.fr (C.D.-L.); 2 CHANEL Fragrance and Beauty, F-93500 Pantin, France; nicola.fuzzati@chanel.com (N.F.); alix.toribio@chanel.com (A.T.); 3 Polymaris Biotechnology, F-29200 Brest, France; bertrand.thollas@polymaris.com (B.T.); anthony.courtois@polymaris.com (A.C.)

**Keywords:** marine bacterial exopolysaccharides, biotechnology, cosmetic industry

## Abstract

Bacteria are well-known to synthesize high molecular weight polysaccharides excreted in extracellular domain, which constitute their protective microenvironment. Several bacterial exopolysaccharides (EPS) are commercially available for skincare applications in cosmetic products due to their unique structural features, conferring valuable biological and/or textural properties. This review aims to give an overview of bacterial EPS, an important group of macromolecules used in cosmetics as actives and functional ingredients. For this purpose, the main chemical characteristics of EPS are firstly described, followed by the basics of the development of cosmetic ingredients. Then, a focus on EPS production, including upstream and downstream processes, is provided. The diversity of EPS used in the cosmetic industry, and more specifically of marine-derived EPS is highlighted. Marine bacteria isolated from extreme environments are known to produce EPS. However, their production processes are highly challenging due to high or low temperatures; yield must be improved to reach economically viable ingredients. The biological properties of marine-derived EPS are then reviewed, resulting in the highlight of the challenges in this field.

## 1. Introduction

Polysaccharides are complex polymers composed of monosaccharides linked by glycosidic bonds, forming large branched or linear molecular structures. These high molecular weight polymers are classified into two groups based on their osidic composition, either homopolysaccharides, containing only one type of monosaccharides, or heteropolysaccharides, consisting of different monosaccharides. The polysaccharide’s primary structure depends not only on chain length (molecular weight can vary significantly from 10,000 to several millions g/mol [1,2,3,4]) or the type of monosaccharides, but also on the linkages between monosaccharides, their sequence and branching pattern, and on substituents [5].

Polysaccharides are ubiquitously found in every living organism from plants to animals, including microorganisms. Regarding microorganisms, two main types of polysaccharides depending on their cellular localization are identified, i.e., intracellular and extracellular polysaccharides, the latter include capsular polysaccharides (CPS), tightly associated with the cell surface and forming a capsule, slime layer loosely associated to cell surface and exopolysaccharides (EPS), excreted by microorganisms into their surrounding environment. EPS can form a slime which remains loosely linked to the cells and can also be dissolved into the extracellular environment. Various microorganisms can produce EPS, including Gram-negative and Gram-positive bacteria, archaea, fungi, and microalgae. Microbial EPS are secondary metabolites, which create a microenvironment around the cells, whose physico-chemical characteristics can balance environmental conditions (pH, salinity, chemicals) that in some cases can be harsh (e.g., deep-sea hydrothermal vents). EPS play a key role in cell protection against dehydration, heavy metals, and other external stress, and may also be involved in aggregation of cells, adhesion onto biotic and abiotic surfaces, biofilm, and nutrient uptake [6]. EPS production by bacteria is an energy-intensive process that accounts for up to 70% of the carbon investment. Despite this high energy cost, EPS benefits are significantly higher, as bacterial growth and survival are increased in their presence [6,7].

Due to their large structural diversity potentially conferring diverse functional properties, and the possibility of production at high scale under controlled conditions, microbial EPS are recognized as important and innovative compounds for the cosmetic field. This review aims to highlight safety and regulatory frameworks of the cosmetic industry, as well as efficacy assessment of actives, candidate-compound development process, and bottlenecks. Based on the scientific literature and patents, we provide an overview of the characteristics of EPS available in cosmetics and their patented applications, focusing on marine-derived EPS, including those produced by extremophiles.

## 2. Chemo-Diversity of (exo)Polysaccharides

Polysaccharides present a huge chemodiversity potential, as 193 billion possible configurations are expected for an oligosaccharide with 6 sugar residues whereas 75 are currently identified. In contrast, there are only 4096 theoretical possible combinations for 6-bp DNA whose structure relies on 4 nucleotide monomer types, and 64 million different peptides composed of 6 amino acids (22 proteinogenic amino acids exist) [8].

EPS display an important diversity in terms of monosaccharide composition, as they can be composed of neutral pentoses (L-arabinose, D-ribose, D-xylose), neutral hexoses (D-glucose, D-galactose, D-mannose, D-allose), deoxyhexoses (L-rhamnose, L-fucose), hexosamines (D-glucosamine, D-galactosamine) and uronic acids (D-glucuronic, D-galacturonic acid). Moreover, sugar residues can be substituted through ester bonds with anionic organic (e.g., lactate, pyruvate, *N* or *O*-Acetate) and/or inorganic (e.g., sulfate) groups. Besides the two configurations of the glycosidic bond involving the anomeric carbon (α and β), linkages between monosaccharides can involve other carbons of the sugars. The anionic groups of substituents together with the carboxylate groups of uronic acids confer a negative charge to the EPS, a critical feature for various physical and biological properties [9,10,11,12]. Linear and branched high molecular weight EPS tend to form helical structures, which may further form double-stranded or triple-stranded structures [13,14,15]. Considering glycosidic linkages, the β-1,4 or β-1,3 linkages between monosaccharides are most commonly found in the rigid backbone of EPS, while the α-1,2 or α-1,6 linkages are typical of the more flexible regions [11]. In addition, the sequence of osidic residues in the polymer chain is important for its functional properties. It can be organized in repeating units composed of up to 8 osidic residues, for an EPS produced by *A. infernus* for example [16].

## 3. Polysaccharides as Skincare Cosmetic Ingredients

The global cosmetic market is continuously growing and is estimated to reach 464 billion USD by 2027, while natural cosmetics are expected to achieve 55 billion USD [17]. Many synthetic ingredients are used in cosmetic products comprising several ingredient types: (1) biologically active ingredients often involved in the product claim, (2) functional ingredients, such as excipients playing the role of vehicle for active ingredients and (3) additives, including preservatives, antimicrobials, perfume, colorants, water, and chelating agents [18].

In recent years, a global tendency has emerged for healthier, more environmentally sustainable, and ecologically friendly products, leading to higher investments in the research and development of innovative natural extracts by cosmetic companies [19]. In response to the increasing demand of naturality by consumers, companies interest increases for bio-based ingredients that can be obtained using green solvents and eco-friendly processes [20]. Bioactive natural products are usually extracted from terrestrial and marine plants as well as microorganisms. However, biotechnology-derived compounds have reproducible composition and structural features, independently of season and climate [19]. Polysaccharides exhibit unique functional properties interesting for cosmetic applications, including gelling, thickening and emulsifying properties. Long dominated by polysaccharides of terrestrial or marine plants, the market has also opened to bacterial EPS and new carbohydrates are continuously under investigation as the polysaccharide market is still expanding. Bacterial EPS comply with the required degree of purity for high value market applications. In cosmetic formulations, polysaccharides are also used as actives, due to their biological properties. Market demand is mainly focused on molecules acting on aging process such as anti-wrinkle agents, molecules which reduce skin thickness and improve skin elasticity, moisturizing compounds, anti-acne molecules, skin depigmentation compounds, UV filters, anti-inflammatory compounds, antioxidants, as well as cytoprotective agents [18,19]. Polysaccharides promote skin feeding, regeneration and maintenance. They account for more than 20% of the molecules used in cosmetics, presenting mainly moisturizing, smoothing, anti-wrinkle activity, whitening, cell repair or regeneration activities, or UV protection [21]. Polysaccharides can be used as humectants to promote a moist environment for the skin [21].

## 4. Regulation and Safety of Cosmetic Products

Specific attention to safety, efficacy, and quality should be paid in cosmetic product development depending on the chosen product category including oral care, skin care, sun care, hair care, decorative cosmetics, body care and perfumes. A cosmetic product can only be launched on the market if it fulfills the targeted country requirements. Cosmetic regulations vary between geographic areas leading to difficulties to ensure compliance in all countries at once. The main regulatory aspects concern cosmetic product definition and classification, pre-market requirements, ingredient management, general labelling, claims concerning advertisement and commercial practices, animal testing, and marketing bans on cosmetic products [22]. Various restrictions in the use of ingredients, classifications of cosmetics and different labelling requirements make the cosmetic regulations more complex [18].

One of the early steps of ingredient and cosmetic product development consists in looking for allowed ingredients in the targeted countries. The EU, China and Japan provide lists for positive, prohibited and restricted products. United States and Canada establish only the list of prohibited and restricted ingredients [18,22]. The EU bans more than 1400 dangerous chemicals, while the USA ban less than 20 ones. Approved ingredients are listed in Annexes of regulation N°1223/2009 and in the European database for cosmetic ingredients (CosIng) [23]. The main specificity of the Chinese regulation is the distinction between “existing” and “new” cosmetic ingredients, including natural or artificial ingredients used in a cosmetic product. Existing ingredients are listed in the Inventory of Existing Cosmetic Ingredients of China, all others are considered as new ingredients [22].

Cosmetic product definitions according to the countries present slight differences concerning functions of the product, parts of the human body chosen, mode of application, indication of use, claims and consumer perspectives [22,24]. Regulation also stipulates that all cosmetics must be manufactured in accordance with the harmonized standards laid out in good manufacturing practices (GMP). These GMP ensure that all products are prepared in a clean environment without any risk of contamination during production [25]. In the USA, regulations are ruled by the Food and Drug Administration (FDA), the two main laws being the Federal Food, Drug and Cosmetic Act (FD&C Act) and the Fair Packaging and Labeling Act (FPLA), while in China, three major authorities are involved in cosmetic product regulation: the State Administration for Market Regulation, the National Medical Products Administration, and the General Administration of Customs. It should be noticed that in addition to country regulations for cosmetic products, attention should be paid to the natural resources used in products. The access to natural resources is regulated by the Convention on Biological Diversity under the Nagoya protocol depending on countries’ decisions [26,27].

In addition, the declaration of ingredients in final cosmetic products must be in accordance with the International Nomenclature of Cosmetic Ingredients, the INCI System [25]. The INCI Nomenclature report from the Personal Care Products Council is available online and presents the different rules for INCI names [28]. The Personal Care Products Council (PCPC) based in the USA and founded in 1894 is the leading national trade association representing cosmetics and personal care products companies. The INCI Nomenclature Program aims to develop unique, informative, standardized accepted names for cosmetic ingredients. INCI names are created by the International Nomenclature Committee (INC) and then published in the International Cosmetic Ingredient Dictionary and Handbook. Biotechnology-derived ingredients are named based on specific conventions which can be applied for bacterial EPS. The first rule concerns high purity molecules: “If a component of the fermentation process has been isolated and purified to a significant extent (>80% based on dry weight), and analytical evidence is provided, the name for one or more of the components may be used (e.g., Sodium Hyaluronate)”. The second rule is applied to well-known end product: “when the end product produced from a given “ferment” or “culture” has a common or usual name, such name may be used (e.g., Xanthan Gum)”. When the end product is not a common name, the following rule is applied: “the product is named using the genus of the microorganism followed by a slash, and the name of the substrate (if applicable), followed by the word “ferment”. Typical fermentation substrates (i.e., glucose, peptone) are not included as part of the INCI name”. Additional terms are included in the name when the products derived by fermentation are further processed by extraction (extract) or filtration (filtrate), or when cells are lysed (lysate) [28]. Despite the complexity of cosmetic regulation, industries have managed to overcome differences to avoid non-compliance of their products and they can still improve and adjust [22].

Safety is a key point in the development of a cosmetic product, for all types of ingredient origin, including marine-derived compounds. Safety of a cosmetic product relies on the safety of all its ingredients evaluated by toxicological assays. Using biotechnology-derived ingredients in a cosmetic product requires additional specific data, such as the sourcing organism’s description, their pathogenicity, toxicity of the produced metabolites, and the fate of viable organisms in the environment [29]. According to the EU regulation N°1223/2009, safety must be evaluated by an independent safety assessor with demonstrated qualification. A cosmetic product safety report (CPSR) is mandatory for every cosmetic product and must be included in the Product Information File (PIF). EU addressed risk assessment guidelines for the cosmetic safety evaluation through the Scientific Committee on Consumer Safety (SCCS). Minimal safety requirements include acute toxicity, corrosivity and irritation, skin sensitization, dermal/percutaneous absorption, repeated dose toxicity, and mutagenicity/genotoxicity. Additional tests may be required, such as reproduction toxicity, carcinogenicity, toxicokinetic studies and photo-induced toxicity. Human response assessment data are very useful when the toxicological profile of the ingredients is safe, although the use of human volunteers can be of great ethical concern [30]. Ingredient safety evaluation comprises a last phase dedicated to the determination of the margin of safety (MoS), taking into account the lowest no observed adverse effect kevel (NOAEL), the dermal absorption value, and the calculated exposure level [30]. Safety studies in EU are carried out using alternative methods validated by the European Center for Validation of Alternative Methods (EURL-ECVAM), as animal testing is prohibited since 2009 for acute toxicological end points; this forbidding was extended in 2013 to repeat-dose toxicity, sensitization, reproductive toxicity, carcinogenicity, and toxicokinetic [25]. Animal testing ban concerns all of the products developed in Europe or intended to be marketed in Europe. The US government also promoted the use of modern technologies to replace animal testing since 2007 and eight states banned animal testing. Toxicology studies are shifting from observation of effects in animals to the assessment through in vitro and in silico tests to predict potential adverse effects in humans [22,30,31].

The Cosmetic Ingredient Review (CIR) is composed of an industry-funded panel of scientific and medical experts, who review and assess the safety of various cosmetic ingredients, including some bacterial polysaccharides [22,32]. In particular, the CIR panel declared that various polysaccharides are nontoxic and safe for the use in cosmetic formulations. Examples of reviewed bacterial polysaccharides include hyaluronic acid and its salts, xanthan gum, gellan gum, wellan gum, levan, dextran, pullulan, bacterial cellulose, and *Pseudoalteromonas* EPS [33,34,35,36]. These positive evaluations strengthen the potential of bacterial polysaccharides in cosmetic products.

## 5. Production and Purification Processes for Bacterial EPS

A major step in developing bacterial-derived ingredients for cosmetic applications relies on production and purification processes. EPS production is dependent on various parameters and process optimization is a key factor for further industrial applications. EPS production involves a common process regardless the targeted development field; examples of EPS production for cosmetics and their specific features are presented in Section 5.3.

### 5.1. Upstream Process

#### 5.1.1. Impact of Culture Conditions on EPS Production

During bacterial culture, production of EPS depends on physicochemical growth conditions, such as nutritional (carbon and nitrogen sources, mineral requirements) and environmental (temperature, pH, dissolved oxygen, salinity, agitation rate) parameters, that greatly affect yield, type, size, and purity of EPS. It is known that several stressing factors can help to increase EPS production (pH or temperature shift, osmotic stress, factors decreasing growth) [37,38,39]. Growth medium composition is a key point to optimize polymer production. Carbon and nitrogen source types and ratios are the main key points for EPS production, as greater amount of carbon tends to increase EPS production, while excessive amounts of nitrogen tend to increase the production of other polymers, such as polyhydroxyalkanoates or degrading enzymes; this is unfavorable for EPS production. Consequently, high amounts of carbon source and low amounts of nitrogen should be provided [40]. Therefore, the carbon to nitrogen (C/N) ratio, defining the proportion of carbon and nitrogen sources should be high, the most suited C/N ratio being 10:1 in the case of *E. aerogenes* [41]. Limitation of some nutrients can also increase the EPS production (phosphorus, sulfur, potassium) [42]. The medium broth is usually supplemented with carbohydrates, such as glucose, sucrose, maltose, lactose, and starch; mannitol and glycerol uses were also reported [37,43,44]. Medium supplementation with vitamins, amino acids, precursors can also stimulate bacterial growth and EPS synthesis [40]. Peptones containing amino acids or ammonium salts are used as nitrogen source [43]. For cosmetics, sugars and peptones of vegetable origin are recommended. Growth conditions do not change significantly the carbohydrate chemical structure, however its content in substituent groups can vary and impact polymer properties [40]. In addition, the molecular weight of EPS, impacting their functional properties and potentially their industrial value, may be affected by culture conditions. For example, high carbon concentrations resulted in increased production yield of high molecular weight diabolican (HE800 EPS), an EPS produced by the deep-sea hydrothermal vent bacterium *V. diabolicus* CNCM I-1629 [43].

Fermentation process induces high costs, due to the use of complex media containing expensive nutrients such as yeast extracts, peptones and salts that can account for 30% of the total cost [45]. The use of industrial byproducts as substrates for the process optimization is an interesting, natural and low-cost alternative for the development of cost-effective process. For now, byproducts can be found in two physical states: liquid resources for classical fermentation (e.g., syrups, molasses, juices) or solid resources for solid-state fermentation (e.g., lignocellulosic biomass, pomaces). However, byproduct sources display some limitations. Indeed, due to their complex composition, different metabolic pathways can be activated, resulting in different polymers synthesized. Therefore, the use of byproducts requires intensive research on pretreatment and downstream processes to guarantee their quality and reproducibility [45]. Some components may also accumulate, inhibit the growth, and lower the production yield. It is Important that good quality substrates are used for high value applications to limit the risk of impurities [40]. Various other parameters also affect EPS production, such as the incubation temperature, which should ideally be below optimal temperature for cell growth to promote EPS synthesis [9]. Concerning pH, it should be kept constant during the fermentation process, ideally between 5.5 and 7.5 (acidic or neutral), depending on the bacterial strains used [46]. High aeration supplies oxygen to the cultures, thus favoring the EPS production. Indeed, aerobic bacteria use molecular oxygen as the final electron acceptor in the electron transport reaction, which becomes a substrate for respiration and energy production, being crucial for cell growth and EPS production [37]. In fermenters, agitation rate, airflow, type and geometry of the stirrer influence dissolved oxygen tension, thus regulating aeration and EPS production [47]. Moreover, EPS production induces great changes in the rheological properties of the medium that may become highly viscous, causing problems of mixing, heat transfer, and oxygen supply [48]. The viscosity increase during production can negatively affect the oxygen distribution in the medium, thus influencing the EPS synthesis and quality. The fermentation is usually stopped to recover EPS when carbon source is totally consumed, often occurring after 48 h.

#### 5.1.2. Optimization of EPS Production

Once a polymer is selected for further investigation, its production must be optimized to achieve high yields. Optimization of medium composition and physicochemical conditions for EPS production requires several experiments. Traditional process development relies on strain and medium screening in batch microtiter plates to study growth parameters by the method of “one factor at a time”; followed by screening in batch shake flask cultures; then bench top bioreactors with online monitoring and control capabilities prior to larger scale validation and optimization to pilot and production scale.

More recent approach relies on the use of statistical approaches, such as designs of experiments (DOE) which are extensively used to decrease time and costs by reducing drastically the number of experiments. For EPS production, common approaches include Plackett-Burman design to identify main factors for process variables, and response surface methodology (RSM) to improve the product yield; most of them are based on the central composite design [43,49]. Other approaches were reported, such as orthogonal matrix and full factorial design [46]. Accelerated process development is a high throughput technique using the combination of DOE and the use of microbioreactor tools with online monitoring and controlled conditions to reduce the number of steps by maximizing process insight in the early phase, and to reduce the need for shake flasks and bench-top bioreactors. Then, conditions are validated in scale bioreactors to pilot and production scale. Microbioreactors have a volume of 0.5 to 15 mL and are equipped with sensors for online monitoring [50].

High throughput techniques examples imply the use of robotic mini bioreactor platform to perform a large number of automated experiments in parallel with advanced feeding strategies similar to industrial process [51]; the use of 4 stirred-tank bioreactors on a L-scale combined to 48 parallel stirred-tank bioreactors on a mL-scale with custom-made transfer reservoirs [52]; or a specific high throughput screening platform designed for EPS production which combines strain cultivation in 96-well format and fast carbohydrate analysis via liquid chromatography coupled with ultra violet and electrospray ionization ion trap detection in 96-well format to detect different sugars, sugar derivatives and substituents such as pyruvate [53].

Other recent advances rely on the use of “-omics”, a set of tools and methods used for metabolic engineering and synthetic biology. Metabolic engineering aims to optimize metabolic pathways for the microbial synthesis of a compound of interest of a specific organism, from a substrate, while generating the maximum production rate and avoiding inhibitors and conditions affecting the growth rate. Metabolic fluxes manipulation is based on databases, libraries of components and growth conditions. Metabolic engineering was already used to optimize hyaluronan, alginate and some other EPS production (*Lactococcus* sp., *Sphingomonas* sp., *Streptococcus* sp.), allowing to obtain higher yields and lower synthesis costs compared to natural production of these strains [54,55]. Synthetic biology aims to assemble genetic pathways in heterologous hosts [56]. However, recombinant technologies for compound production in a more efficient heterologous host are highly challenging for heteropolysaccharides and are not easily acceptable in the cosmetic field, making the cost objectives difficult to achieve.

#### 5.1.3. EPS Production at Large Scale

EPS production at large scale includes several steps, such as bacterial cultivation in a bioreactor, followed by cell removal, extraction, and purification of EPS from the culture supernatant, and drying or dilution (downstream process). Industrial production can involve bioreactors with volumes ranging from 500 L to 50,000 L [20]. For bacterial cells, EPS biosynthesis is a high energy-consuming pathway since 70% of cell energy is dedicated to this pathway [6]. The production of EPS occurs at a specific phase of the bacterial growth depending on the microorganism used. It mainly takes place during the late log phase of the growth and continues during stationary phase reaching the maximal production in this phase [3,57,58] which is usually reached after 24 h of incubation. On the other hand, some microorganisms synthesize EPS during exponential phase [59].

### 5.2. Downstream Process

When the production of EPS in bioreactor is completed, downstream process determines the purity of excreted polymers. An efficient downstream process should be developed to increase EPS recovery yield and its purity, while decreasing production cost and environmental impacts. The purification process can greatly affect the feasibility of the entire process, since the extraction step accounts for 50% of the total process cost [37]. In addition, optimization is often needed for each polymer by considering the EPS physicochemical characteristics and the final requirements.

If the polymer is thermally stable, an additional step can be conducted at the end of fermentation before cell removal. Heat treatment of the broth at 90–95 °C during 10–15 min can be applied to kill bacterial cells and inactivate degrading enzymes. However, heat treatment can cause polymer degradation, which reduces broth viscosity [40]. Moreover, high temperature can also cause cell lysis resulting in the release of potential contaminants from bacterial cells difficult to separate. Basic recovery processes involve cell removal by centrifugation and/or filtration, then the polymer is precipitated using alcohols (e.g., ethanol, isopropanol) in the presence of salt and subsequently dried using freeze-drying or drum dying [40]. The elimination of biomass is a key point to guarantee safety and purity of EPS. Centrifugation is commonly used to separate cells from culture medium containing soluble EPS, before microfiltration step. At the lab scale, high speed centrifugation is needed to remove cells from supernatant (10,000 to 20,000 g). Although high speed has no major impact on EPS integrity, it may cause cell lysis leading to the presence in broth of impurities, such as cell debris, proteins, membrane constituting components such as lipopolysaccharides (LPS) [20]. Therefore, for high purity grade polymers needed for cosmetics, additional steps should be included to eliminate most impurities in the final products (cell debris, salts and proteins). In particular, anionic EPS, characterized by the presence of uronic acids or sulfate groups, have the ability to bind several compounds, such as proteins. Additionally, if some residual carbon substrate is still present in the culture medium at the end of the fermentation process, it can be co-extracted too [60]. Several additional steps can increase polymer purity, such as reprecipitation of the polymer from diluted aqueous solution (<1 g/L), chemical addition (e.g., salting out or protein precipitation with trichloroacetic acid), enzymatic deproteinization using proteases or membrane filtrations. However, the use of chemicals can greatly alter the polymer. The most suited technique to increase the polymer purity remains membrane processes, in particular ultrafiltration, widely used in the downstream purification. Membranes composed of polyethersulfone (PES) with a cut-off between 50 to 300 kDa are usually used. Purification process is followed by the EPS drying to obtain the polymers in “ready-to use” form.

**Figure 1:**
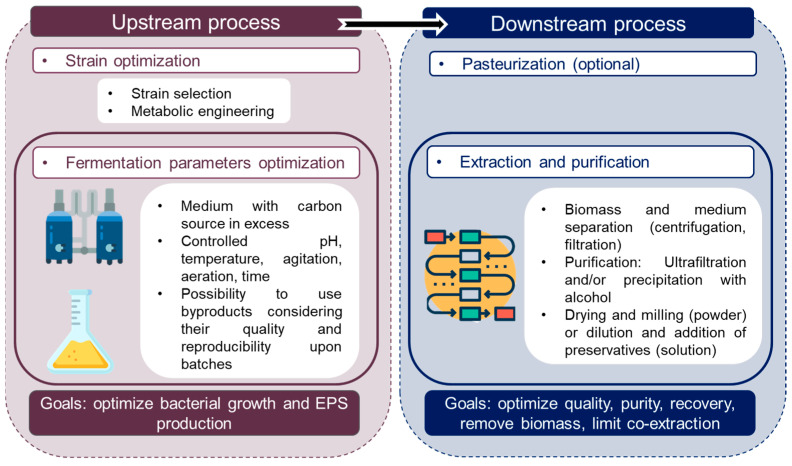
Key factors for EPS production including upstream and downstream processes. Icons from Freepik [61].

Dried EPS are obtained using freeze drying, which is easily feasible at lab scale but too expensive at an industrial scale. Spray drying, a cheaper technique, can be used for large scale production, even if adjuvants such as maltodextrin should be added to overcome high viscosity problems encountered during concentration. Otherwise, EPS solubilization in water can be more difficult to obtain after complete drying. Concerning polymer conservation before final use, dried form is the best suitable to prevent microbial contamination and polymer degradation. Aqueous solutions of EPS can also be chosen if cosmetic approved preservatives are used to prevent microbial contamination [20]. The final choice for downstream process to obtain pure EPS is a compromise between product recovery, quality, purity and impact on polymer properties [40], considering costs. Key factors of upstream and downstream processes are presented in Figure 1.

### 5.3. Bacterial EPS Production for Cosmetic Ingredients Development

In this review, we intended to focus on bacterial EPS and reviewed several patented marine-derived EPS for cosmetic applications. These data were extracted from a patent review on Orbit Express database (Questel©); we focused on patents deposited by cosmetic companies or suppliers. Several examples of production and purification process were given in these patents. Some of these examples were selected and are presented in Table 1, with information including medium and carbon source used, incubation conditions (temperature, pH and time), and methods of extraction and purification. Xanthan production by *X. campestris* described in patents and literature is highlighted, for example the production medium contains soy proteins, calcium carbonate, and corn syrup as a carbon source. Usual production process of xanthan relies on broth pasteurization at the end of fermentation, cell removal, purification by ethanol precipitation, drying, and milling. For marine bacteria, all media contain sea salts, artificial seawater or seawater, required for marine bacteria growth. A salt solution composed of sodium chloride, magnesium chloride, magnesium sulfate, potassium chloride, calcium chloride, sodium bicarbonate, sodium bromate, and iron chloride can also be used [4].

Medium often contains vegetal peptones, allowing for the use of non-GMO (Genetically Modified Organism) and non-animal compounds. Yeast extracts are added to provide vitamins, enzymatic cofactors and micronutrients essential to growth. Carbon sources used for EPS production are mainly sugars: glucose, galactose, mannose, cellobiose, maltose, starch, glycogen, lactose, as well as mixtures [62]. Disaccharides and polysaccharides as carbon sources are not suitable for all bacterial strains. In particular, those devoid of enzymes to degrade the carbon source cannot produce EPS. Even if lactose is considered as an ideal feedstock due to its low price and abundance [37], its animal origin is a great concern for cosmetic applications. In addition, only strains able to produce degrading β-galactosidase enzyme can metabolize lactose [63]. Glucose is often the preferred carbon source due to its wide availability through industrial production from sugar beets with possibility of controlled and known supply chains. Bacteria cells removal after fermentation is achieved using centrifugation from 2000 to 10,000 g during 2 min to 1 h (Table 1). Filtration process is then conducted using 0.2 to 0.7 µm filters to eliminate residual bacteria and agglomerates. Purification by ultrafiltration on 10 to 300 kDa cut-off membranes is usually performed. Some patents mentioned dialysis with 10 kDa membranes for EPS of *Halomonas* sp. [4,62]. However, this process is only suitable at lab scale, not for industrial purposes. Drying process is carried out by freeze-drying, also suitable at lab scale but too expensive at industrial scale. When EPS are obtained with desired purity, they can be dried to obtain pure powders [4,64,65], sometimes followed by additional dilution in demineralized water at 0.5 to 2% with addition of preservatives to Inhibit bacterial contamination [64].

**Table 1:** Examples of EPS production conditions from both cosmetic patents and scientific literature: bacterial strain, medium, carbon source, fermentation conditions (temperature, pH, time), extraction and purification. Production yields are not available in patents.

Strain	Medium	Carbon Source	Temperature, pH, Time	Extraction & Purification	Refs.
*X. campestris*	Soy proteins 2.5 g/L, calcium carbonate 0.1 g/L	Corn starch 38 g/L Glc, Suc 20–40 g/L	28–30 °C; pH 6.0–7.5 60–100 h	Broth pasteurization at 90–120 °C for 20 min, cell removal, purification by ethanol precipitation, drying, milling.	[66,67,68]
*C. marina* CNCM I-4353	Peptone 4 g/L, yeast extract 1 g/L, salts 27 g/L	Glc, Suc or Fru 30 g/L	25–30 °C	Cell removal by centrifugation and filtration, extraction by ultrafiltration using 300 kDa membranes.	[64]
*H. anticariensis* LMG P-27891	Yeast extract, malt extract, peptone 0.5–10 g/L, salts	Glc 25 g/L	32 °C; pH 7.0; 24–72 h	Cell removal by centrifugation and filtration, ultrafiltration, dialysis using 10 kDa PES membranes. Purification by ethanol, acetone or isopropanol precipitation.	[62]
*H. eurihalina* LMG P-28571	Yeast extract 3 g/L, malt extract 3 g/L, pea peptone 5 g/L, salt solution	Glc 10 g/L	32 °C; pH 7.0; 40 h	Cell removal by centrifugation and filtration, ultrafiltration 10 kDa cut-off PES membranes or dialysis with 10 kDa cut-off membrane. Purification by ethanol, acetone or isopropanol precipitation.	[4]
*Pseudoalteromonas* sp. CNCM I-4150	Medium with salts	Glc 20 g/L	29 °C; pH 7.5; 72 h	Cell removal by centrifugation and filtration, purification with distilled water by ultrafiltration on 100 kDa cut-off PES membranes.	[69]
*V. alginolyticus* CNCM I-5035	Zobell medium: sea salts 30 g/L, Yeast extract 1 g/L, Peptone 4 g/L	Glc 30 g/L	25 °C; pH 7.2; 72 h	Cell removal by centrifugation and filtration, ultrafiltration using 100 kDa cut-off membranes.	[65]
*Vibrio* sp. CNCM I-4239; *Vibrio* sp. CNCM I-4277	Zobell medium with salts	Glc 20 g/L	29 °C; pH 7.5; 72 h	Cell removal by centrifugation and filtration, purification with distilled water by ultrafiltration on 100 kDa cut-off PES membranes.	[70,71]

Glucose (Glc), Sucrose (Suc), Fructose (Fru).

## 6. EPS for Cosmetic Applications: Non-Marine versus Marine EPS

### 6.1. Non-Marine Bacterial EPS in Cosmetics

**Figure 2:**
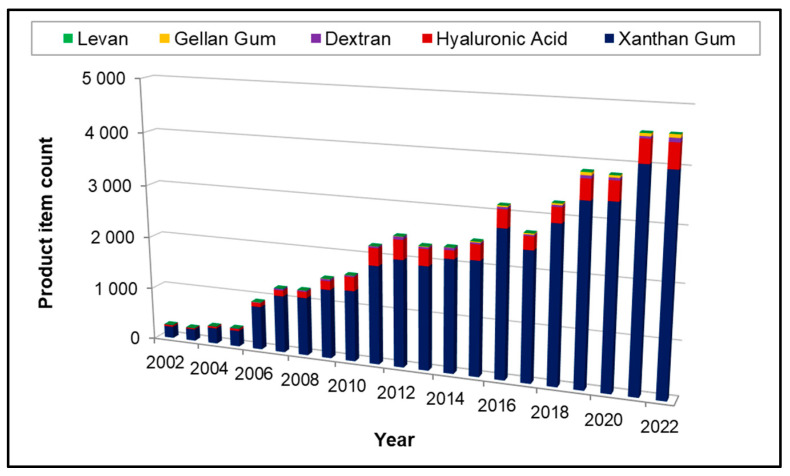
Evolution of new skincare products containing bacterial EPS over 20 years on three markets: France, USA and China, according to a statistical study retrieved from the Mintel’s Global New Products Database (GNPD, Mintel Group Ltd., London, UK) on 18 October 2023 [72]. Product item count corresponds to filed product sheets (one product per country). INCI names are listed in increasing total product items over 20 years: levan (34), gellan gum (342), dextran (661), hyaluronic acid (4764) and xanthan gum (39,613).

Over the past 20 years, the number of new cosmetic products containing bacterial EPS increased significantly, as demonstrated by a statistical study retrieved from the Mintel’s Global New Products Database (GNPD) on skincare products on three markets (France, USA, China) (Figure 2). Five specific INCI names were targeted, corresponding to bacterial EPS: levan, gellan gum, dextran, xanthan gum, and hyaluronic acid. We assumed that hyaluronic acid was mainly of bacterial origin. Statistical study demonstrated that these EPS are mainly used in face and neck care (50% of total skincare products). Xanthan gum is widely used as a functional ingredient in skincare products on these three markets (39,613 total items). The use of hyaluronic acid as active constantly increases to reach 4764 total items in skincare. In contrast, dextran, gellan gum and levan use in skincare products is considerably lower with total number of items over 20 years of 661, 342, and 34, respectively.

**Figure 3:**
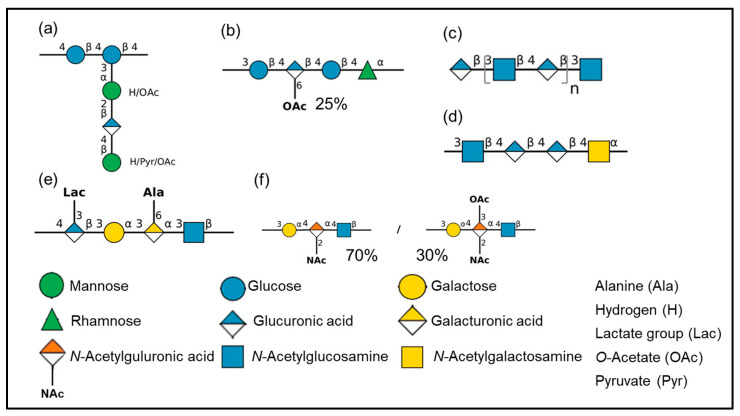
EPS structure examples, created with Drawglycan-SNFG [91,92] using standardized glycan symbols. (**a**) Xanthan gum produced by *X. campestris* [74,75], (**b**) Gellan gum produced by *P. elodea* [80], (**c**) Hyaluronic acid [85], (**d**) EPS diabolican produced by *V. diabolicus* CNCM I-1629 [93], (**e**) EPS produced by *V. alginolyticus* CNCM I-5034 [94], (**f**) EPS produced by *V. alginolyticus* CNCM I-5035 (Epidermist 4.0^TM^) [65].

Xanthan gum is the most extensively used non-marine bacterial EPS in cosmetics since its discovery and first commercial development by Kelco [73]. Xanthan is an anionic high-molecular weight (0.4–15 × 10^6^ g/mol) heteropolysaccharide secreted by *Xanthomonas* sp. strains, usually industrially obtained from *X. campestris*. It is composed of a pentasaccharide repeating unit with a cellobiose backbone and a trisaccharide side chain containing one glucuronic acid between two mannose residues, substituted by pyruvyl and acetyl groups (Figure 3a) [74,75]. Side chains account for 65% of the molecular weight of xanthan and play a significant role in the molecular conformation [76]. Xanthan undergoes conformation transitions from helix to random coil depending on stimuli such as pH, ionic strength, temperature and shear [77]. At low concentrations and low shear rate (pseudoplastic behavior), xanthan displays the unusually high viscosities important to its suspension-stabilizing properties. Low temperature and high salt concentration favor ordered helix forms, while high temperature and low salt concentration favor disordered coil shapes [78]. Due to its outstanding solution properties, xanthan gum is widely commercially used for a wide range of applications in the food, pharmaceutical, and cosmetic industries [1,79].

Another example of EPS largely used in cosmetic products constitutes gellan gum. This linear anionic high-molecular weight (0.24–2.2 × 10^6^ g/mol) heteropolysaccharide, being a part of the “sphingan” polymer family, is produced by *Pseudomonas* sp. and *Sphingomonas* sp. bacterial strains, and is composed of rhamnose, glucose and glucuronic acid, substituted by acetyl groups (Figure 3b) [80]. Gellan has interesting functional properties due to its ability to form a transparent gel in the presence of divalent cations, resistant to acid and heat [81]. Slightly acylated gellan forms hard and brittle gels, while highly acylated gellan forms soft and elastic gels [82]. In cosmetic products, it is used as thickening agent and emulsion stabilizer [33].

Hyaluronic acid (HA) or hyaluronan is another well-known anionic high-molecular weight (2 × 10^6^ g/mol) polysaccharide belonging to GAG family used in cosmetics. It was firstly discovered in the vitreous humor of the eye [83]. HA is recognized as an important moisturizer due to its high water retention capacity, being able to bind 1000 times its volume in water [84]. It is mainly used in cosmetic products as skin conditioning and viscosity increasing agent [29]. This linear polymer is based on the repeating disaccharide unit composed of glucuronic acid and *N*-Acetylglucosamine (Figure 3c) [85]. Several bacteria are also able to produce HA, amongst *Streptococcus* sp. [86], a genus which unfortunately comprises pathogenic bacteria [87]. To encounter this issue, heterologous production was achieved in bacteria belonging to ‘generally recognized as safe’ (GRAS) group. *B. subtilis*, a GRAS bacterium, was thus engineered for HA production [54]. In cosmetics, HA is often used as an anti-aging or anti-wrinkle agent [88], promoting skin hydration and elasticity [89]. HA is widely used as a dermal filler and replaced collagen-based dermal fillers [84,90].

Several other EPS have been described for cosmetic applications, additional examples including cellulose, dextran, Fucogel (Solabia), FucoPol, GalactoPol and levan are given in Table 2. Even though cellulose and dextran have the same osidic composition (glucose), their glycosidic linkages differ, as monosaccharides are linked through β-1,4 linkages and through α-1,6 linkages, respectively. In consequence, these EPS display distinct structural and conformational features. Cellulose is known for its crystalline appearance and insolubility in aqueous solvents, bacterial cellulose is known for its higher water holding capacity, higher crystallinity and higher purity compared to plant derived cellulose [95], while dextran is highly soluble in water. Concerning high fucose containing EPS, Fucogel (Solabia) displays interesting bioactivities such as anti-aging properties, probably arising from its anionic charges, its linear structure and its lower molecular weight (4 × 10^4^ g/mol) compared to other presented EPS. On the other hand, FucoPol has a branched structure, possesses anionic charges and a higher molecular weight (2–6 × 10^6^ g/mol) conferring functional and bioactive properties. Otherwise, GalactoPol is a linear and anionic high molecular weight EPS (>1 × 10^6^ g/mol), mainly composed of galactose and three other neutral sugars (mannose, glucose and rhamnose), substituted with three anionic groups (succinate, pyruvate, acetate) exhibiting functional properties. Finally, levan is a linear homopolysaccharide composed of fructose, which can be linear or branched depending on the producing strain, of high molecular weight generally around 2 × 10^6^ g/mol, with rheological and film forming properties, as well as some bioactivities.

It appears that non-marine bacteria produce a diversity of EPS owing functional and/or bioactive properties making them suitable candidates as cosmetic ingredients. Their anionic nature, related to the presence of uronic acids and substituting groups as well as their high-molecular weights seem to determine their valuable functional properties. Some EPS, such as xanthan or HA have been extensively studied since their early discovery and are now widely used in the cosmetic industry.

### 6.2. Marine Bacterial EPS in Cosmetics

With ocean representing 70.8% of the Earth surface and the existence of a large number of niches characterized by specific conditions, this wide ecosystem still remains underexplored and represents a source of new biodiversity and chemical diversity. Many industry fields exhibit great interest to these potentially new compounds, including bacterial EPS. In particular, marine bacteria constitute a rich source of innovative EPS, that are explored for their skincare effects in cosmetic products. The main described bacteria are Gammaproteobacteria belonging to the genera *Alteromonas*, *Pseudoalteromonas* and *Vibrio* [3,59,93,129,130,131,132,133]. *Alteromonas* and *Pseudoalteromonas* species usually produce highly branched anionic EPS composed of both neutral sugars and uronic acids substituted with sulfate, pyruvate and/or lactate groups. In contrast, *Vibrio* species synthesize linear anionic EPS containing mainly uronic acids and hexosamines, and can also be substituted with acetyl and/or lactate groups as well as amino acids [12,60,94,134]. The presence of anionic monosaccharides and different negatively charged substituting groups is determinant for functional properties of marine EPS.

A non-exhaustive list of marine EPS with cosmetic applications and their characteristics is presented in Table 3. These data were extracted from a patent review made on Orbit Express database (Questel©), focusing on patents deposited by cosmetic companies or suppliers. Some other data for these patented EPS were also extracted from articles published in peer-reviewed scientific journals. Presented patents are granted alive to date (database accessed on March 2023), except for patents related to the HYD657 (Abyssine^TM^ PF) EPS produced by *A. macleodii* subsp. *fijiensis* biovar deepsane CNCM I-1285, which have expired. Eleven EPS are shown, including data information on producing bacterial strain, EPS composition and molecular weight, as well as scopes of cosmetic actions and summarized bioactivities. It appears that marine-derived EPS are only used as active ingredients. The eleven presented polymers encompass two strains of *Alteromonas* sp., one *Cobetia marina* strain, two strains of *Halomonas* sp., one *Pseudoalteromonas* sp., and four strains belonging to *Vibrio* genus (Table 3). The strain *C. marina* CNCM I-4353 is outlined twice in the table as its EPS is patented in native form and as depolymerized derivative, depicting different molecular weights and bioactivities [64,135]. Another depolymerized derivative from *V. alginolyticus* CNCM I-4151 is also discussed [136].

Abyssine^TM^ PF produced by the deep-sea hydrothermal vent bacterium *A. macleodii* subsp. *fijiensis* biovar deepsane (HYD657 CNCM I-1285) was firstly developed by Lanatech and is now commercialized by Lucas-Meyer [27]. Abyssine^TM^ PF is an anionic heteropolysaccharide of high-molecular weight (1 × 10^6^ g/mol) composed of neutral sugars (galactose, glucose, mannose, fucose), uronic acids (glucuronic acid, galacturonic acid), and substituted with three anionic groups (sulfate, lactate, pyruvate). The EPS is known for its soothing properties and it also reduces irritation and provides photoprotection from UVB irradiations [138]. *Halomonas* sp. produces an EPS mainly composed of neutral sugars, however, uronic acids and substituting sulfate groups may also be present at low levels (e.g., *H. eurihalina* LMG P-28571) [4]. Similar osidic composition was also reported for EPS produced by *Pseudoalteromonas* sp. CNCM I-4150 [69]. Other EPS are substituted with various anionic organic and inorganic groups and some of them are also substituted with amino acids. For example, *C. marina* CNCM I-4353, which produces a sulfated EPS composed of neutral sugar, uronic acid and hexosamine residues, is additionally decorated with amino acids (threonine and serine) [64]. The native high-molecular weight (1 × 10^6^ g/mol) EPS exhibits anti-inflammatory properties, whereas its low molecular weight derivatives (2 × 10^5^ g/mol) possess anti-aging properties and improve skin appearance, and barrier functions.

EPS synthesized by *Vibrio* sp. are linear anionic polysaccharides devoid of sulfate mainly composed of uronic acids and hexosamines that may also be substituted with amino acids. For instance, a high molecular weight (1 × 10^6^ g/mol) EPS, diabolican, produced by *V. diabolicus* CNCM I-1629 (Figure 3d) displays original structural features close to those of hyaluronic acid as it is composed of glucuronic acid and *N*-Acetylglucosamine as well as *N*-Acetylgalactosamine [93]. The EPS produced by *V. alginolyticus* CNCM I-5034 (Figure 3e) is composed of glucuronic acid, *N*-Acetylglucosamine, galactose and galacturonic acid and it is substituted with alanine and lactate groups [94]. Epidermist 4.0^TM^ produced by *V. alginolyticus* CNCM I-5035 (Figure 3f) is a linear EPS composed of galactose, *N*-Acetylglucosamine and *N*-Acetylguluronic acid, with 30% of acetyl groups [65].

To modulate the EPS bioactivities, depolymerization can be performed to reduce the molecular weight of the native polymer. Several techniques are used to decrease the molecular weight of the polymer, such as chemical depolymerization using free radicals or acids [145,146], as well as mechanical [147] or ultrasonic degradations [148]. Free-radical depolymerization with hydrogen peroxide and copper, used as a metal catalyst, is reproducible and can be controlled through pH regulation and adjustment of hydrogen peroxide concentration, leading to low molecular weight derivatives between 20,000 and 100,000 g/mol [146,149]. Two patented derivatives were obtained using radical depolymerization of the native EPS from *Pseudoalteromonas* sp. CNCM I-4150 and *Vibrio* sp. CNCM I-4239 strains [69,71] (Table 4). Even if depolymerization using acids or free radicals is suitable to decrease the molecular weight of polysaccharides, this method is not specific and may lead to the loss of substituents or sugar residues. Enzymatic hydrolysis remains more specific and more sustainable for polysaccharide depolymerization. However, it is highly challenging due to specific EPS structures, which implies the use of appropriate enzymes. Indeed, it was shown that enzymatic depolymerization of infernan (GY785 EPS) produced by the deep-sea hydrothermal vent bacterium *A. infernus* was not effective although various commercially available enzymes were tested. Only intracellular protein extract of this bacterium, containing a polysaccharide lyase, was able to depolymerize the EPS that it produces [16,150]. Another patented depolymerization process which was successfully applied to prepare low molecular weight derivatives is based on supercritical fluid-accelerated hydrothermolysis [151]. It allows partial depolymerization without altering monosaccharide pattern, decorating amino acids and sulfate groups. This technique using carbon dioxide heated to 200 °C and high pressures up to 250 bars was applied to depolymerize native EPS from *C. marina* CNCM I-4353 [64,135] and *V. alginolyticus* CNCM I-4151 [136].

Several non-marine and marine bacterial EPS are available on the market as cosmetic ingredients. Table 4 presents examples of non-marine bacterial EPS, such as xanthan, gellan and Fucogel^®^ together with their trademarks as well as marine bacterial EPS and their main ingredients suppliers. These commercial EPS can be used by cosmetic companies to develop new skincare products.

### 6.3. EPS-Producing Extremophilic Bacteria

EPS-producing marine bacteria were isolated from different environments, including atypical ones presenting extreme conditions such as the Antarctic marine environment, sea ice, marine snow, microbial mats, hypersaline environments, shallow and deep-sea hydrothermal vent environments [58,60,130]. Some of the marine bacteria producing the EPS presented in Section 6.2 were isolated from extreme environments, such as *A. macleodii* subsp. *fijiensis* biovar deepsane (HYD657 CNCM I-1285) [129] and *V. diabolicus* CNCM I-1629 [93], both isolated from the polychaeta annelid *Alvinella pompejana* living close to hydrothermal vents located on the East Pacific Rise [160].

In addition, *C. marina* strains, psychrophilic bacteria evolving in cold water, were isolated from coastal sea samples [161] and mussels [162]. *C. marina* KMM 296 could even grow in the wide range of temperatures from 4 to 42 °C [162,163]. *H. eurihalina* is a moderately halophilic bacterium which can spread in diverse saline environments, such as solar salterns, intertidal estuaries, hydrothermal vents, hypersaline lakes and open ocean [163,164]. *H. eurihalina* MS1 isolated from saline soil in Alicante (Spain) was shown to be an EPS producing strain [165,166]. Another *Halomonas* sp., *H. anticariensis* strains FP35 and FP36 isolated from saline soils (Spain) also produced EPS [165,166]. *Pseudoalteromonas* strains are only found in marine environments, they possess environmental adaptation capacities as they can survive in extreme habitats, such as hydrothermal vents and polar areas [130,132]. Moreover, *V. alginolyticus* is a halophilic bacterium growing in the ocean or estuary environment [167]. These marine bacteria can grow in harsh conditions, and EPS production is important for their survival. Although isolated from extreme environments, these strains’ physiological requirements and tolerances are compatible with classical conditions of production, i.e., mesophilic temperature and neutral pH. These production conditions can be kept upon scaling up of an industrial process.

On the other hand, true marine extremophiles have also been described to produce EPS; their growth optima are extreme (high temperature, low temperature for example) (Table 5). As marine bacteria usually require 10 to 30 g/L salts to grow, they are considered as moderate halophiles. The EPS presented in Table 5 emphasize the wide diversity of EPS among marine bacteria and the potential of extremophilic bacteria, however they were not studied for their cometic applications. Concerning their chemical diversity, marine-derived EPS have similar composition independently on their extremophilic origin, while the bacterial genus is the key factor to classify EPS diversity [60]. As indicated in Table 5, *C. psychrerythraea* 34H produces an EPS containing *N*-Acetyl-quinovosamine (QuiNAc) which has not been identified previously (Section 6.2); other monosaccharides are encountered in non-marine and marine-derived EPS.

Commercial production of marine bacteria relies on the ability of the strain to grow in classical conditions of production, as high or low temperatures induce high energy demand and require specifically designed bioreactors, and salt concentration needs to be decreased to limit equipment corrosion. Bacteria growing at extreme values of pH require the use of acids and alkalis that induce corrosion and are risky to handle at industrial scale. Moreover, the use of piezophiles cannot be considered as it requires specific and costly equipment associated with high risks due to high pressure. However, among marine extremophiles, only thermophilic and psychrophilic bacterial strains have been reported to produce EPS (Table 5). None of them are currently commercialized or have been considered as actives for cosmetics.

EPS production yield in marine thermophiles is lower than mesophilic marine strains [168]. Requirement of high temperature also imposes shake flasks as bioreactors are not developed for such high temperature. Fermentation for psychrophiles is longer than mesophiles. All of these specificities may hinder development of EPS from extremophiles unless more efforts are conducted for optimizing fermentation process conditions including further developments for scale-up production of EPS in bioreactor.

**Table 5:** Examples of EPS produced by marine extremophilic bacteria: bacterial strain, type and environment, production conditions and yield, EPS composition (monosaccharides and substituting groups), molecular weight (Mw). Not indicated (N.I).

Bacterial Strain	Type	Environnent	In-Lab Production Conditions and Yield	EPS Composition	Mw (g/mol)	Refs.
*Bacillus licheniformis* T14	Thermophilic Halophilic	Marine hydrothermal vent	Bioreactor: 50 °C; pH 8.0; 48 h; NaCl 50 g/L Yield: 366 mg/L	Fru, Fuc, Glc, GalN, Man	1 × 10^6^	[169,170]
*Bacillus licheniformis* B3-15	Thermophilic	Shallow hydrothermal vent	Shake flask: 45 °C Yield: 165 mg/L	Man, Glc	6 × 10^5^	[171]
*Geobacillus thermodenitrificans* sp. B3-72	Thermophilic	Shallow hydrothermal vent	Shake flask: 65 °C; 72 h Yield: 70 mg/L	Man, Glc	4 × 10^5^	[172]
*Geobacillus* sp. 4004	Thermophilic	Marine hot spring	Bioreactor: 60 °C; pH 7.0; 28 h Yield: 90 mg/L	Man, Glc, Gal, GlcN, Ara	1 × 10^6^	[173]
*Rhodothermus marinus* DSM 4252 and MAT 493	Thermophilic	Shallow marine hot spring	Shake flask: 65 °C; 48 h Yield: N.I	Ara, Xyl Acetate, sulfate	8 × 10^4^	[174]
Strain 4001	Thermophilic	Marine hot spring	Shake flask: 65 °C; 48 h Yield: 60 mg/L	Man, Glc, Gal, ManN	4 × 10^5^	[175]
*Colwellia psychrerythraea* 34H	Psychrophilic	Arctic sediments	Shake flask: 4 °C Yield: N.I	GalA, QuiNAc Alanine decoration	N.I	[176]
*Colwellia* sp. GW185	Psychrophilic	Antarctic sponge	Shake flask: 15 °C; 168 h Yield: 183 mg/L	Glc, Man, Gal, GalN, GlcA, GalA	N.I	[177]
*Marinobacter* sp. W1-16	Psychrophilic	Antarctic seawater	Shake flask: 15 °C; 192 h Yield: 139 mg/L	Glc, Man, Gal, GalN, GalA, GlcA Sulfate	3 × 10^5^	[178]
*Polaribacter* sp. SM1127	Psychrophilic	Arctic brown algae	Shake flask: 15 °C; 120 h 2110 mg/L	GlcNAc, Man, GlcA	2 × 10^5^	[179]
*Pseudoalteromonas* sp. MER144	Psychrophilic	Antarctic seawater	Shake flask: 4 °C; 336 h Yield: 318 mg/L	Glc, Man, GlcN, Ara, GlcA, GalA, Gal	3 × 10^5^	[180]
*Pseudoalteromonas* sp. SM20310	Psychrophilic	Arctic sea ice	Shake flask: 15 °C; 72 h Yield: 567 mg/L	Man, Glc, Gal, Rha	2 × 10^6^	[181]
*Pseudoalteromonas* sp. SM9913	Psychrophilic	Deep-sea sediment	Shake flask: 15 °C; 52 h Yield: 5250 mg/L	Glc, Ara	4 × 10^4^	[182]
*Pseudomonas* sp. ID1	Psychrophilic	Antarctic marine sediment	Shake flask: 11 °C; 120 h Yield: N.I	Glc, Gal, Fuc	2 × 10^6^	[183]
*Shewanella* sp. CAL606	Psychrophilic	Antarctic sponge	Shake flask: 4 °C; 240 h Yield: 329 mg/L	Glc, Gal, Man, GalN, GlcA, GalA	N.I	[177]
*Winogradskyella* sp. CAL396	Psychrophilic	Antarctic sponge	Shake flask: 4 °C; 240 h Yield: 397 mg/L	Man, Ara, GalA, GlcA, Gal, Glc, GlcN	N.I	[177]
*Winogradskyella* sp. CAL384	Psychrophilic	Antarctic sponge	Shake flask: 4 °C; 240 h Yield: 144 mg/L	Glc, Man, GalA, Ara, Gal, GlcN, GlcA	N.I	[177]

Arabinose (Ara), Fructose (Fru), Fucose (Fuc), Galactose (Gal), Galacturonic acid (GalA), Galactosamine (GalN), Glucose (Glc), Glucuronic acid (GlcA), Glucosamine (GlcN), *N*-Acetylglucosamine (GlcNAc), Mannose (Man), Mannosamine (ManN), *N*-Acetyl-quinovosamine (QuiNAc), Rhamnose (Rha), Xylose (Xyl).

EPS are produced in response to biotic and abiotic stresses, and their secretion is one of the mechanisms used to tolerate harsh conditions including cold stress and ice crystal damage for psychrophiles, high temperature for thermophiles eventually exposed to large temperature gradients, high salts for halophiles, and acidic or toxic metal-containing environments for acidophiles or metal resistant microorganisms [184,185,186]. Some microorganisms also have the ability to thrive in environments with multiple extreme conditions, such as deep-sea hydrothermal vents with intermittent extreme physical and chemical gradients between vent fluids and surrounding seawater, or Kopara that are Polynesian microbial mats found in pools of Polynesian atolls and subjected to variations in salinity, dessication and sun exposure. This protective role of EPS relies on the formation of a mucous slime around bacterial cells and is claimed in the bioactivities of final ingredient, emphasizing their biomimetic action on skin, including protection against low-temperature for psychrophiles [179,187], chelation of trace metals and binding of heavy metals [177,188] although activity features of EPS from extremophiles were not studied for cosmetics.

However, extremophilic bacteria represents a new biodiversity source of EPS, strengthening their chemical diversity.

### 6.4. Bioactivity Evaluation of Marine EPS

Marine-derived EPS presented in Section 6.2 exhibit interesting bioactivities for cosmetic applications. They are further detailed in this section. Examples of assays used to demonstrate these bioactivities are also presented, they can be applied to various active candidates depending on molecular weight, solubility and targeted bioactivities.

Due to regulatory requirements and safety assessments, EPS bioactivities need to be demonstrated on skin models for their commercial development. The EPS presented in Table 3 possess interesting biological activities, which were assessed using in vitro and ex vivo techniques. Examples of targeted bioactivity assays among selected EPS are shown in Table 6. These EPS possess anti-aging [62], anti-inflammatory [136] and anti-acne [136] properties as well as moisturizing [70] and slimming effects [62]. They promote vascularization [64] and improve skin barrier function [71,136]. The first step in assessing the EPS bioactivities is to determine the cytotoxicity of the compound on the selected cell culture or skin model, to verify that the tested compound can be applied at a suitable concentration without adverse effects. Cytotoxicity data are not systematically given in patents. However, cell viability is often assessed using MTT (3-(4,5-dimethylthiazol-2-yl)-2,5-diphenyltetrazolium bromide) or AlamarBlue assays.

Typically, two-dimensional (2D) and three-dimensional (3D) cell culture models are used to further assess the potential activities of the EPS. For instance, the EPS from *H. anticariensis* LMG P-27891 was applied in 2D cell culture of dermal fibroblasts to assess its effect on type I collagen synthesis [62]. Similar 2D model was used to demonstrate moisturizing properties of the EPS from *Vibrio* sp. CNCM I-4277, which was shown to increase HA synthesis [70]. 2D cell culture of keratinocytes was selected to assess the barrier function of the skin upon treatment with EPS from *Vibrio* sp. CNCM I-4239 [71]. A particular example of 2D cell culture of subcutaneous pre-adipocytes was used to assess the potential slimming effect, i.e., the decrease of lipid accumulation, in the presence of EPS produced by *H. anticariensis* LMG P-27891 [62]. 2D cell co-cultures composed of dermal fibroblasts and vein endothelial cells were used to study more complex processes, such as the formation of blood vessels (angiogenesis), to trigger the vascularization stimulation effect of EPS from *C. marina* CNCM I-4353 [64].

More complex 3D cell culture models were also used to demonstrate EPS properties, such as reconstructed human skin. Specific reconstructed human skin with induced inflammation or aging was used to assess the potential anti-inflammatory activities of the EPS produced by *V. alginolyticus* CNCM I-4151. Inflammation was studied by measuring the expression of metalloproteinase 3 (MMP3), the level of which increases in the case of acne lesions. EPS from *V. alginolyticus* CNCM I-4151 on this model led to decreased MMP3 levels. Moreover, the effect of this EPS on reconstructed aged human skin improved barrier function by increasing the expression of proteins of the stratum corneum [136].

Skin explant, an ex vivo skin model, was also used to demonstrate EPS bioactivities. For example, skin explants exhibiting induced inflammation by the addition of LPS were used to study the anti-inflammatory activity of the EPS from *V. alginolyticus* CNCM I-4151. An inflammatory cytokine known to stimulate sebum production in the skin, Interleukin-8 (IL-8) was quantified, its production level decreased after treatment with the studied EPS [136]. Furthermore, a high molecular weight EPS produced by the deep-sea hydrothermal vent bacterium *V. diabolicus* CNCM I-1629 was shown in a dermal equivalent model (composed of collagen I and EPS and containing living human dermal fibroblasts) to promote both collagen structuring and fibroblast colonization (migration and proliferation) with an extracellular matrix synthesis by the cells [143,144]. As an active ingredient, Epidermist 4.0^TM^ improves skin barrier functions and increases skin defense against pathogens involved in acne [65]. These examples emphasize the interesting bioactivities of some marine bacterial EPS and demonstrate their value as cosmetic active ingredients.

### 6.5. Structure-Function Relationship

It was shown through the cited examples that bacterial EPS present a wide diversity not only in terms of structural features but also of molecular weights, which further determine their functional properties relevant for cosmetic products. Anionic high molecular weight xanthan and gellan gums are mainly used as functional ingredients providing textural properties to cosmetic formulations, while HA constitutes a multifunctional ingredient as it possesses both textural and bioactive properties. Marine bacterial EPS are mainly used for their various biological activities, which make these polymers good candidates as active ingredients. However, structure-function relationships of EPS for cosmetic applications are not easy to identify due to important diversity of their compositions and structures (if known), their molecular weights and molecular conformations. Nevertheless, some hypotheses can be proposed. Due to their anionic nature resulting from the presence of negative charges of uronic acids and sulfate, acetate or pyruvate groups, EPS can efficiently bind positively charged components (e.g., proteins, growth factors) through ionic interactions. Such a role is largely known for non-sulfated (hyaluronic acid) and sulfated (heparan, heparan sulfate, chondroitin sulfate) GAG of mammalian tissues. Indeed, through interactions with multiple proteins, GAG regulate cellular processes (adhesion, migration, proliferation, differentiation) and are thus involved in physiological processes [189]. These interactions have been shown important for skin repair or regeneration activities, where the presence of EPS was shown to stimulate the synthesis of the extracellular matrix rich in HA and collagen by the cells (Table 3 and Table 6).

Polysaccharides in cosmetic formulations are also used to maintain skin structural integrity and health due to their moisturizing, soothing, and anti-wrinkle activities, as well as whitening action and UV protection. Since polysaccharides are highly hydrophilic polymers, their moisturizing properties result from their high-water binding capacity due to the hydrogen bond formation between their multiple polar groups and water molecules, which confers moisturizing activity and further prevents from the loss of water from the skin surface [21]. The presence of different functional groups in the EPS structure (hydroxyl, sulfate, carboxyl, carbonyl, secondary amine) also provides metal chelating activity, an important property for protective effect against pollution [190,191]. Skin whitening is an important cosmetic market in Asia and is mainly due to inhibition of tyrosinase activity involved in melanin biosynthesis. This property has been discussed in relation of mannose presence in polysaccharide extracts [192]. Polysaccharides endowed with anti-oxidant, in particular through reactive oxygen species (ROS) scavenging [21], or anti-inflammatory activities are valuable in anti-aging application, and UV protection of skin. Besides its whitening action, mannose was also shown to inhibit inflammatory damage in skin [192]. However, the underlying structural basis of action mechanisms is still limited.

Biological activities of bacterial EPS on skin also depend on the molecular weight of polymers, i.e., the length of the polysaccharide chain, and its molecular conformation. Similarly to other polysaccharides from plant and animal origin, bacterial EPS are highly flexible macromolecules adopting helical conformations in solution [14,193,194]. With increasing length, polysaccharide chains display tendency to high entanglement reinforced by hydrophilic and ionic interactions. Therefore, low molecular weight polymers can easier cross the skin barrier and activate various biological pathways (e.g., anti-aging), compared to high molecular weight native polymers, acting at the epidermis level through moisturizing, barrier function and anti-wrinkle smoothing effects. Regarding human skin, hyaluronic acid penetration was shown to depend on the molecular weight, as HA of high molecular weight (1000 to 1400 kDa) remained at the surface of the stratum corneum, and HA of low molecular weight (20 to 300 kDa) was able to crosse the stratum corneum [195].

**Figure 4:**
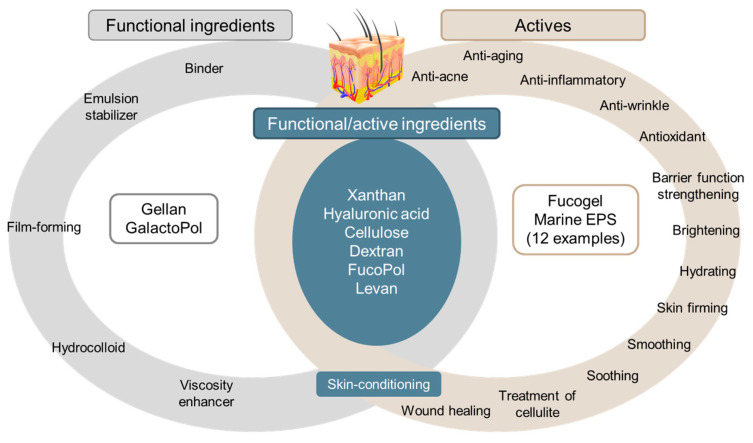
Functional and bioactive properties of marine and non-marine bacterial EPS.

Functional and active properties of non-marine and marine EPS are presented in Figure 4. Skin conditioning agents include various ingredients acting as emollient, humectant, skin appearance improver, occlusive. For this reason, even if skin conditioning agents are not strictly considered as actives, they can contribute to skin protection and are used in product formulation (e.g., water retention). Therefore, they are classified at the interface between functional ingredients and actives. Xanthan, hyaluronic acid, Fucogel^®^ and levan act as skin conditioning agents in addition to their functional and bioactive properties.

## 7. Challenges and Perspectives

**Figure 5:**
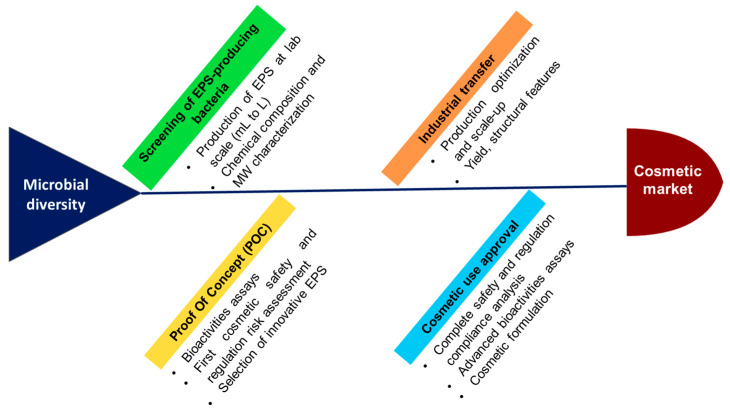
Marine bacterial EPS development process for cosmetic products: from microbial diversity to the cosmetic market.

In conclusion, bacterial EPS constitute renewable, biodegradable, and biocompatible compounds endowed with unique functional and bioactive skincare properties that can be exploited in cosmetic products. EPS produced by non-marine bacteria are used for their rheological properties and some bioactivities as well, while marine-derived bacterial EPS are mainly used for their bioactive properties. Development of EPS from bacterial origin for cosmetics includes several steps, presented in Figure 5, from microbial diversity to cosmetic market, with four main steps: (1) screening of EPS-producing bacteria, (2) Proof Of Concept (POC), (3) industrial transfer, and (4) cosmetic use approval. Success of the development of innovative EPS use in cosmetics can be successfully achieved by considering both regulatory framework and safety requirements early in the process. Companies introduce the risk management associated with new product development at the beginning of their research programs to establish a well-defined strategy and to mitigate the risk of failing. The main challenges for EPS development at industrial scale also rely on production costs. In the personal care industry, special attention must be paid to the cost of compound production since technology and manufacturing processes with poor yields can be too expensive, leading to economically unviable products. Since the use of complex media induces high costs (30% of global cost), the use of less expensive substrates (e.g., byproducts) and various optimization steps, allowing to increase the production yield, should be considered. This requires further improvements of upstream and above all downstream processes since extraction and purification of polymers still account for an important part of the production costs (50%). Specific attention should also be paid to water and power consumption. Therefore, efforts should be focused on developing cheaper and reproducible processes while leading to a final product with high purity and quality. Similar bottlenecks are encountered in the development of bacterial EPS, regardless their origin. From a global perspective, production process is strain and EPS dependent. However, marine bacteria culture implies the use of salts, whose concentration should be decreased to limit equipment corrosion. Regarding safety, attention should be paid to EPS production and purity, as potentially co-extracted compounds, such as proteins or lipopolysaccharides could induce toxic effects. Thus, appropriate toxicologic assays should be performed to assess the product safety. In addition, the status of intellectual property and regulatory requirements among countries must be determined to ensure EPS development as cosmetic ingredients.

In the future, together with metabolic engineering, the use of recombinant strains could lead to increased production yields with highly efficient processes obtained by genetically-engineered strains. However, to allow commercial development of products, heterologous production should meet regulatory framework and safety requirements; therefore, a particular attention should be paid to this point. As EPS biosynthesis is a complex pathway, the use of genetic engineering is quite challenging, especially in the case of heteropolysaccharides, requiring the involvement of several enzymes organized in large genetic clusters which include also specific regulation genes. Success of EPS production by recombinant strains will also depend on the consumer perception/acceptance. Indeed, naturality concept for ingredients gains more and more attention for consumers. The way in which the regulation framework will evolve about genetically modified microorganisms (GMM), and how the companies will communicate and popularize the use of GMM will shape the future of cosmetic industry.

Nowadays, the impact of ingredients on the environment is another great concern. Methods to monitor this impact are still under development. Companies often use life cycle analysis (LCA), aiming to ensure social, economic, and environmental equilibrium of the product from the supply chain to wastes.

**Figure 6:**
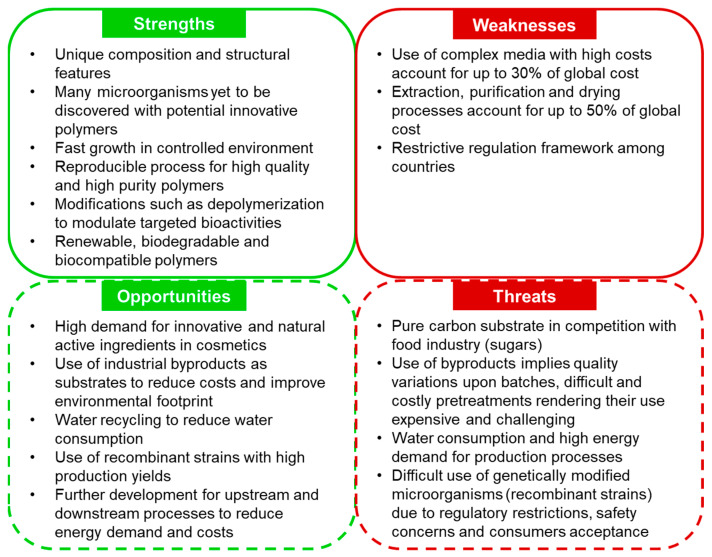
SWOT analysis for bacterial EPS use in cosmetics.

The present review seeks to highlight both main points and challenges of process development for EPS production (Figure 5) from microbial diversity to the cosmetic market. As a consequence, marine-derived EPS represent real opportunities for cosmetic industry. Strengths, Weaknesses, Opportunities and Threats (SWOT) analysis of bacterial EPS in cosmetics, presented in Figure 6, highlights their huge potential and point out the key challenges that remain to be overcome.

## Figures and Tables

**Table 2:** Non-marine bacterial EPS extensively used in cosmetics: bacterial EPS, producing strain, EPS composition (charges, ramifications, monosaccharides and substituting groups), molecular weight (Mw) and functional properties.

Bacterial EPS	Bacterial Strain	EPS Composition	Mw (g/mol)	Functional Properties	Ref.
Xanthan	*Xanthomonas* sp.	Anionic, branched Glc, Man, GlcA, Pyruvate, acetate	0.4–15 × 10^6^	Hydrocolloid, binder, emulsion stabilizer, viscosity enhancer, thickening agent Skin conditioning agent	[1,33,73,74]
Gellan	*Sphingomonas* sp.	Anionic, linear Glc, Rha, GlcA Acetate, glycerate	0.24–2.2 × 10^6^	Hydrocolloid, emulsion stabilizer, viscosity enhancer	[33,80,96,97]
Hyaluronic acid (HA)	*Streptococcus* sp.	Anionic, linear GlcA, GlcNAc	2 × 10^6^	Viscosity enhancer, high water retention capacity Skin conditioning agent Bioactive: anti-wrinkle, moisturizing, skin elasticity enhancer, dermal filler	[34,84,85,86,88,89,98]
Cellulose (β-glucan)	*Aliivibrio* sp., *Agrobacterium* sp., *Gluconacetobacter* sp., *Komagataeibacter* sp., *Pseudomonas* sp., *Rhizobium* sp.	Neutral, linear Glc	1 × 10^6^	Insoluble in aqueous solvents, highly crystalline, high degree of hydration, emulsion stabilizer Bioactive: moisturizer	[95,99,100,101,102,103,104,105]
Dextran	*Lactobacillus* sp., *Leuconostoc* sp., *Pediococcus* sp., *Streptococcus* sp., *Weissella* sp.	Neutral, linear Glc	2–40 × 10^6^	Binder, bulking agent Bioactive: skin smoothing, brightening agent, anti-inflammatory	[106,107,108,109,110,111,112]
Fucogel	*Klebsiella* sp.	Anionic, linear Fuc, Gal, GalA Acetate	4 × 10^4^	Skin conditioning agent Bioactive: skin moisturizing, anti-aging	[113,114]
FucoPol	*Enterobacter* A47	Anionic, branched Fuc, Gal, Glc, GlcA Succinate, pyruvate, acetate	2–6 × 10^6^	Hydrocolloid, emulsifying, flocculating and film-forming agent Bioactive: antioxidant, wound healing, photoprotection	[115,116,117,118,119,120]
GalactoPol	*Pseudomonas* sp.	Anionic, linear Gal, Man, Glc, Rha Succinate, pyruvate, acetate	1–5 × 10^6^	Hydrocolloid, emulsifying, flocculating and film-forming agent	[121,122]
Levan	*Aerobacter* sp., *Bacillus* sp., *Halomonas* sp., *Pseudomonas* sp., *Streptococcus* sp., *Zymomonas* sp.	Neutral, linear or branched Fru	2 × 10^6^	Water-soluble, strongly adhesive, film former, viscosity enhancer Skin conditioning agent Bioactive: anti-inflammatory, cell proliferative	[2,33,123,124,125,126,127,128]

Fructose (Fru), Fucose (Fuc), Glucose (Glc), Glucuronic acid (GlcA), *N*-Acetylglucosamine (GlcNAc), Galactose (Gal), Galacturonic acid (GalA), Mannose (Man), Rhamnose (Rha).

**Table 3:** Marine bacterial EPS with cosmetic applications from literature and patent review: bacterial strain, EPS composition (charges, monosaccharides and substituting groups), molecular weight (Mw) (* depolymerized EPS), scopes of action and bioactivities.

Bacterial Strain	EPS Composition	Mw (g/mol)	Scopes of Action	Bioactivities	Refs.
*A. macleodii* subsp. *fijiensis* biovar deepsane HYD657 CNCM I-1285	Anionic Gal, Glc, Rha, GlcA, GalA, Man, Fuc Sulfate, lactate, pyruvate	1 × 10^6^	Soothing Irritation	Soothing effect; reduction of sensitive skin irritation by chemical, mechanical and UVB aggression; promotion of skin repair.	[129,137,138,139,140,141]
*Alteromonas* sp. CNCM I-4354	Anionic GlcA, Glc, Gal, GalA, Man	1 × 10^6^	Wrinkles	Wrinkle depth reduction; collagen fibers contraction inducing a tensing effect.	[142]
*C. marina* CNCM I-4353	Anionic Glc, Rha, Gal, GlcA, GalA Sulfate	1 × 10^6^	Soothing Inflammation	Inhibition and prevention of inflammation.	[135]
*C. marina* CNCM I-4353	Anionic Glc, Rha, GlcNAc, GalA, Gal Sulfate 2 amino acids (threonine and serine)	2 × 10^5^ *	Barrier function Skin appearance Aging	Improvement of barrier function and moisturizing of the skin in the treatment of aged skin; improvement of skin repair kinetics against external aggressions.	[64]
*H. anticariensis* LMG P-27891	Neutral or anionic Man, Rha, Glc Optional: GalA, Xyl	1 × 10^4^	Inflammation Aging Wrinkles Skin firming	Treatment of cellulite; reduction of skin lipid accumulation; stimulation of lipolysis and collagen synthesis; reduction of the amount of nocturnin in cells.	[62]
*H. eurihalina* LMG P-28571	Neutral or anionic Glc, GlcN, Man, Rha, Gal Optional: Fuc, GlcA Sulfate	1 × 10^4^	Aging Wrinkles Skin firming	Promotion of collagen synthesis and connexins levels.	[4]
*Pseudoalteromonas* sp. CNCM I-4150	Anionic Glc, Gal, GlcA, GlcNAc, GalA, Man	8 × 10^5^ *	Aging Wrinkles	Improvement of skin moisturizing due to the water retention capacity.	[69]
*V. alginolyticus* CNCM I-4151	Anionic GalA, GlcNAc 2 amino acids (alanine and serine)	2 × 10^5^ *	Aging Inflammation Acne	Reduction of inflammation reduced; improvement of quality of the superficial layers of the epidermis; degradation of the extracellular matrix reduced.	[136]
*V. alginolyticus* CNCM I-5035	Anionic Gal, GlcNAc, GulNAcA	5 × 10^5^	Barrier function Acne	Improvement of physical and chemical barriers function by increasing the keratinocyte differentiation and epidermal renewal. Increasement of immune defense against pathogens involved in acne.	[65]
*Vibrio* sp. CNCM I-4239	Anionic GlcNAc, GlcA, GalNAc	1 × 10^5^–1 × 10^6^ *	Hydration Inflammation	Promotion of the healing process; inhibition of neuronal exocytosis (inflammation; acne; wrinkle reduction).	[71]
*Vibrio* sp. CNCM I-4277	Anionic GlcA, GlcNAc, Glc, Fuc Sulfate	1 × 10^6^	Aging Wrinkles	Increase of hyaluronic acid synthesis.	[70]
*V. diabolicus* CNCM I-1629	GlcA, GlcNAc, GalNAc	1 × 10^6^	Skin regeneration	Collagen structuring and extracellular matrix establishment by dermal fibroblasts	[93,143,144]

Fucose (Fuc), Glucose (Glc), Glucuronic acid (GlcA), Glucosamine (GlcN), *N*-Acetylglucosamine (GlcNAc), Galactose (Gal), Galacturonic acid (GalA), *N*-Acetylgalactosamine (GalNAc), *N*-Acetylguluronic acid (GulNAcA), Mannose (Man), Rhamnose (Rha), Xylose (Xyl).

**Table 4:** Examples of bacterial EPS trademarks as cosmetic ingredients (non-exhaustive list).

EPS	Examples of Trademarks	Refs.
Non-marine bacteria	Xanthan: Keldent^®^, Keltrol^®^ (CP Kelco, Atlanta, GA, USA), Rheocare^®^ (BASF, Ludwigshafen, Germany), Satiaxane (Cargill, Minneapolis, MN, USA), Safic’ Care T XG 80^®^ (Safic Alcan, Puteaux, France) Gellan: Kelcogel^®^ (CP Kelco, Atlanta, GA, USA) Fucogel^®^ (Solabia, Pantin, France)	[152,153,154,155,156]
Marine bacteria	Epidermist 4.0^TM^, EPS Bright^TM^, EPS Seafill^TM^, EPS Seamat^TM^, EPS Seaglow^TM^, EPS Seapur^TM^ (Codif Technologie Naturelle, Saint-Malo, France) Hyadisine^®^, Hyanify^®^ (Lubrizol, Wickliffe, OH, USA) Abyssine^TM^ PF, Exo-H^TM^, Exo-P^TM^, Exo-T^TM^ (Lucas-Meyer, Québec, QC, Canada)	[157,158,159]

**Table 6:** Examples of biological activities of marine bacterial EPS: bacterial strain, claims, cell culture models (two-dimensional, 2D and three-dimensional, 3D) as well as assessed activity and the method used.

Strain	Claims	Cell Culture Model	Activity and Analysis	Refs.
*C. marina* CNCM I-4353	Vascularization	Co-culture of human dermal fibroblasts (NHDF) and human umbilical vein endothelial cells (HUVECs) infected with a lentivirus that express green fluorescent protein	Quantification of angiogenesis (fluorescence levels expressed by HUVECs)	[64]
*H. anticariensis* LMG P-27891	Anti-aging	Human dermal fibroblasts (2D)	Type I collagen synthesis (ELISA assay)	[62]
*H. anticariensis* LMG P-27891	Slimming	Human subcutaneous pre-adipocytes in a complete differentiation medium (2D)	Reduction of the lipid accumulation “adipogenesis” (fluorescence assay)	[62]
*V. alginolyticus* CNCM I-4151	Anti-inflammation	Skin explants inflamed by lipopolysaccharides addition	Interleukin production quantification (IL-8 levels of expression)	[136]
*V. alginolyticus* CNCM I-4151	Anti-inflammation Anti-acne	Inflamed reconstructed human skin (3D)	Inflammation level studied by metalloproteinase expression (MMP3 mRNAs levels of expression)	[136]
*V. alginolyticus* CNCM I-4151	Barrier function	Reconstructed aged human skin (3D)	Late Cornified Envelop Proteins (LCEs) proteins of the stratum corneum (gene expression of LCE3)	[136]
*Vibrio* sp. CNCM I-4277	Moisturizing	Human dermal fibroblasts (2D)	Hyaluronic acid synthesis (ELISA assay)	[70]
*Vibrio* sp. CNCM I-4239	Barrier function	Human keratinocytes (2D)	Healing test (microscopic observations of cells compared before and after treatment on the scrap region)	[71]
*Vibrio* sp. CNCM I-4239	Cytotoxicity	Human dermal fibroblasts (2D)	Proliferation assay to measure cell viability (fluorescence assay)	[71]
*V. diabolicus* CNCM I-1629	Promotion of fibroblast proliferation	Dermal equivalent matrices with human dermal fibroblasts (3D)	Proliferation and migration of fibroblasts and production of an extracellular matrix	[143,144]

## Data Availability

No new data were created or analyzed in this study. Data sharing is not applicable to this article.
